# Risk stratification of adult T‐cell leukemia/lymphoma using immunophenotyping

**DOI:** 10.1002/cam4.928

**Published:** 2016-12-30

**Authors:** Huseini H. Kagdi, Maria A. Demontis, Paul A. Fields, Juan Carlos Ramos, Charles R. M. Bangham, Graham P. Taylor

**Affiliations:** ^1^ Section of VirologyDepartment of MedicineImperial College LondonLondonUK; ^2^ Department of HematologyGuys HospitalLondonUK; ^3^ Department of Hematology/OncologyUniversity of Miami School of MedicineMiamiFlorida

**Keywords:** Adult T‐cell leukemia/lymphoma (ATL), chemokine receptor(s), human T‐lymphotropic virus type 1 (HTLV‐1), immunophenotyping, interleukin receptor(s)

## Abstract

Adult T‐cell leukemia/lymphoma (ATL), a human T‐lymphotropic virus type 1 (HTLV‐1)‐associated disease, has a highly variable clinical course and four subtypes with therapeutic and prognostic implications. However, there are overlapping features between ATL subtypes and between ATL and nonmalignant (non‐ATL) HTLV‐1 infection complicating diagnosis and prognostication. To further refine the diagnosis and prognosis of ATL, we characterized the immunophenotype of HTLV‐1‐infected cells in ATL and non‐ATL. A retrospective study of peripheral blood samples from 10 HTLV‐1‐uninfected subjects (UI), 54 HTLV‐1‐infected patients with non‐ATL, and 22 with ATL was performed using flow cytometry. All patients with ATL had CD4^+^ CCR4^+^ CD26^−^ immunophenotype and the frequency of CD4^+^ CCR4^+^ CD26^−^ T cells correlated highly significantly with the proviral load in non‐ATL suggesting CD4^+^ CCR4^+^ CD26^−^ as a marker of HTLV‐1‐infected cells. Further immunophenotyping of CD4^+^ CCR4^+^  CD26^−^ cells revealed that 95% patients with ATL had a CD7^−^ (≤30% CD7^+^ cells), whereas 95% HTLV+ non‐ATL had CD7^+^ (>30% CD7^+^ cells) immunophenotype. All patients with aggressive ATL had a CCR7^+^ (≥30%), whereas 92% with indolent ATL and 100% non‐ATL had a CCR7^−^ (<30%) immunophenotype. Patients with nonprogressing indolent ATL were CD127^+^ but those with progressive lymphocytosis requiring systemic therapy had a CD127^−^ (≤30%) immunophenotype. In summary, HTLV‐1‐infected cells have a CD4^+^ CCR4^+^ CD26^−^ immunophenotype. Within this population, CD7^−^ phenotype suggests a diagnosis of ATL, CCR7^+^ phenotype identifies aggressive ATL, while CCR7^−^ CD127^−^ phenotype identifies progressive indolent ATL.

## Introduction

Human T‐lymphotropic virus type 1 (HTLV‐1) is a complex delta retrovirus infecting an estimated 10 million individuals worldwide. The majority of infection leads to a chronic asymptomatic carrier state (AC) but 2% to 6% develop adult T‐cell leukemia/lymphoma (ATL) and another 3% inflammatory disorders, for example, HTLV‐1‐associated myelopathy (HAM)^1^. The development of ATL occurs almost exclusively in patients with a high HTLV‐1 proviral load (PVL) 2. Indeed in the United Kingdom cohort, incident ATL occurred only among patients with PVL >10% (37% of all patients) with a lifetime risk of 13.3 per 1000 patient‐years 3. These high PVL patients are thus considered high risk and there is a need for better risk stratification and prevention in this group.

The diagnosis of ATL is based on clinical features, morphology (lymphocytes with characteristic “flower cell” morphology), immunophenotyping (CD3^+^ CD4^+^ CD25^+^ CD7^−^), and serological evidence of HTLV‐1 infection 4. However, some of these features are also seen in patients with nonmalignant (non‐ATL) HTLV‐1 infection (i.e., AC and patients with HAM). This makes diagnosis difficult in some cases. ATL has a highly variable clinical course ranging from asymptomatic blood test abnormality to rapidly fatal multisystem disease. Shimoyama et al. first proposed classification of ATL into four subtypes: smoldering, chronic, acute, and lymphoma. Smoldering and chronic ATL were suggested to have an indolent course, while acute and lymphoma an aggressive course 5. The majority of patients with indolent ATL either develop progressive disease or transform to aggressive ATL 6. A classification of chronic ATL into favorable and unfavorable subtypes on the basis of elevated lactate dehydrogenase, blood urea nitrogen, and low albumin, was suggested to predict the risk of progression. Favorable subtypes, at low risk of progression, are managed with active monitoring or topical therapy. Immediate systemic therapy is indicated in unfavorable chronic and aggressive ATL 7. Conventional chemotherapy is frequently ineffective in both indolent and aggressive ATL (7–48% and 11–27% 4 year survival, respectively) 8. A meta‐analysis of retrospectively studied cases indicated that outcomes with zidovudine (ZDV) + interferon‐*α* (IFN) treatment were more successful than chemotherapy in leukemic disease. The outcome was especially good in chronic ATL (100% 5‐year survival) 9. However, the 30% of patients with acute ATL who achieved complete remission with ZDV+IFN also had a better outcome with 82% 5‐year survival. Thus, ATL subtype and treatment response help in predicting long‐term outcomes. However, there remains a wide variation in response within each subtype.

Since most aggressive ATL arise de novo from non‐ATL HTLV‐1 infection, there is an urgent need for better markers to diagnose preclinical ATL in the patients at high risk. In addition, markers to identify indolent disease at risk of progression and treatment refractory disease will help devise best treatment strategies. We hypothesize that the immunophenotype of infected cells in ATL is different from non‐ATL HTLV‐1 and informs prognosis. The aim of this study was detailed immunophenotypic characterization of HTLV‐1‐infected cells in patients with non‐ATL and ATL to improve the diagnosis and prognostication of ATL.

The HTLV‐1 infection burden comprises thousands of small clones in non‐ATL with the emergence of a large leukemic clone in ATL 10, 11. HTLV‐1 viral proteins are not detected in unmanipulated, in vivo‐infected cells, hence aberrant expression of host cell markers are used as surrogate markers of HTLV‐1 infection. The commonly used immunophenotype of ATL cells is CD3^+^ CD4^+^ CD25^+^ CD7^−^. However, cells with this immunophenotype are also present in uninfected healthy individuals and patients with non‐ATL HTLV‐1 infection. To look for patterns associated with ATL and non‐ATL HTLV‐1 infection, the expression of T‐cell‐associated markers including costimulatory molecules (CD4 and CD8), surface markers (CD7 and CD26), interleukin receptors (CD25 and CD127), and chemokine receptors (CCR4 and CCR7) were analyzed. Interleukin 2 (IL‐2) and IL‐7 contribute to activation and homeostatic proliferation of mature T cells via their receptor CD25 and CD127, respectively. Immune T‐cell activation associated with chronic viral infections, including asymptomatic carriage of HTLV‐1, leads to upregulation of CD25 and downregulation of CD127 on T cells 12. ATL cells have been shown to express CD25 as well as secrete IL‐2 leading to auto‐ and paracrine proliferation 13. CCR4 and CCR7 facilitate homing of mature T cells to skin and lymph nodes, respectively. HTLV‐1‐infected cells express CCR4 as well as secrete its ligand MDC to facilitate infection spread 14. CCR4 and CCR7 expression by ATL cells correlates with skin and nodal involvement 15, 16. CD7 is an immunoglobulin superfamily receptor involved in apoptosis regulation and is expressed on normal CD4 T cells. CD7 expression is frequently lost in ATL and is associated with poor prognosis 17. CD26/dipeptidyl peptidase IV (DPPIV), a T‐cell‐activation antigen, is expressed on normal CD4 T cells. CD26 expression is frequently lost in non‐ATL and ATL HTLV‐1 infection by means of epigenetic machinery 18.

## Material and Methods

### Patients and cells

The patient cohort is based at the National Centre for Human Retrovirology (NCHR) at St Mary's Hospital, Paddington, London, UK and University of Miami School of Medicine, Miami, USA. Diagnosis of HTLV‐1 infection, HAM, and ATL was made according to World Health Organization criteria 4, 19. Patients attending the NCHR are invited, regardless of diagnosis and treatment, to participate in a Communicable Diseases Research Tissue Bank approved by the UK National Research Ethics Service (references 09/H0606/106 and 15/SC/0089). Following written informed consent, additional blood samples for HTLV research are obtained during routine venesection for clinical tests. Samples from the patients with ATL were collected at diagnosis or relapse prior to systemic therapy. Samples at University of Miami School of Medicines were collected under IRB‐approved study “Study of Blood, Tissue and Body Fluids of Viral Associated Malignancies” (EPROST No. 20030608). Samples are stored under license in accordance with the Human Tissues Act 2004.

### Baseline clinical characteristics

Patients with HAM as well as AC with high or low HTLV‐1 PVL were selected randomly from the cohort to represent the spectrum of non‐ATL HTLV‐1 infection. Patients with chronic and acute ATL were selected for the study of indolent and aggressive ATL, respectively, as these have circulating malignant cells. The samples from all patients with indolent ATL and four of the patients with aggressive ATL were collected at diagnosis, while the samples from six patients with aggressive ATL were collected at relapse.

Baseline demographic and clinical characteristics of the study population are shown in Table 1. There was no significant difference in age, sex, or ethnicity between AC, patients with HAM, indolent and aggressive ATL. The total lymphocyte count, relative frequency of CD4^+^ T cells, and PVL were significantly higher in patients with indolent and aggressive ATL compared to AC and HAM (*P* < 0.0001). None of the 54 patients with non‐ATL HTLV‐1 infection progressed to overt ATL during a median of 68.5 months observation.

**Table 1:** Baseline patient characteristics

Characteristics	non‐ATL		ATL		Difference between the four groups
(Median; range)	AC	HAM	Indolent	Aggressive	
Numbers	26	28	12	10	NA
Age in years	56.6 (31.4–75.8)	59.8 (23.9–82.4)	55.2 (33.6–75.8)	62.6 (49.4–75.5)	*P* = 0.17
Sex					*P* = 0.06
Male	2	6	4	4	
Female	24	22	8	7	
Ethnicity					*P* = 0.12
Afro Caribbean	20	22	12	10	
others	6	6	0	0	
Lymphocyte count (10^9^/L)	1.7 (1–3.8)	2.1 (1.2–4.3)	10.2 (3.8–77.5)	16.6 (6.5–83)	*P* < 0.0001
CD 4 T cells/ Lymphocytes (%)	50 (24–66)	50 (35–61)	84 (62–97)	86 (72.5–98.1)	*P* < 0.0001
Proviral load (%)	7 (0.001–27.9)	8.5 (0.4–63.3)	53.3 (22.7–177.9)	51.4 (22.6–145.5)	*P* < 0.0001
Systemic ATL therapy	None	None	7^a^	10	NA
Treatment response	NA	NA	CR (6), PR (1) a/b	CR (1)^b^, PR (1)^b^, PD (8)	NA

a One chronic ATL transformed to acute ATL.

b Developed progressive disease within 3 months.

Abbreviations AC: Asymptomatic carrier, HAM: HTLV‐1 associated myelopathy ATL: Adult T‐cell leukaemia/Lymphoma CR: complete remission, PR: Partial remission, PD: Progressive disease, NA: Not applicable.

Of the 12 patients with indolent ATL, 10 had the unfavorable subtype as described above. Five patients with indolent ATL (two favorable and three unfavorable) had stable lymphocytosis after a median of 40 months' follow‐up and did not require systemic ATL therapy. Seven patients required systemic therapy: five had progressive lymphocytosis > 10^10^/L, one had lymphomatous, and one acute transformation at a median of 4.6 months after first assessment. The patients with progressive lymphocytosis (chronic ATL) and lymphomatous transformation achieved complete remission (CR) on treatment with ZDV+IFN and combination chemotherapy, respectively. The patient with acute ATL transformation achieved a short duration partial remission (PR) and is described in detail below.

All 10 patients with aggressive ATL received systemic therapy as first‐ and second‐line treatment. All patients received combination chemotherapy while nine also received ZDV+IFN, respectively. Two patients responded (1 CR, 1 PR) to first‐line treatment but suffered relapse within 3 months, while eight patients had progressive disease. None responded to second‐line treatment.

### Cell storage

Peripheral blood mononuclear cells (PBMCs) were isolated from fresh whole blood by density gradient centrifugation on Histopaque‐1077 (Sigma‐Aldrich, St Louis), washed in phosphate‐buffered saline (PBS, Sigma‐Aldrich), cryopreserved in 10% dimethyl sulphoxide (Sigma‐Aldrich), 90% heat‐inactivated fetal calf serum (FCS) (Gibco, Carlsbad), and stored in liquid nitrogen until use. Cryopreserved cells were thawed by incubation at 37°C for 2 min and washed with cold 1% FCS three times.

### Multi parameter flow cytometry

Thawed cryopreserved PBMCs were incubated sequentially in infrared fixable viability stain followed by fluorochrome‐conjugated monoclonal antibodies listed in Table S1 for 30 min in total at room temperature (RT) at a concentration of 10^6^ cells/50 *μ*L. The cells were then fixed using fixation buffer from Foxp3/Transcription Factor Staining Buffer Set (eBioscience, San Diego) and stored at 4°C in 1% FCS overnight until analysis on Becton Dickinson LSR II or Fortessa II. For Ki67 staining, the fixed cells were washed with permeabilization buffer followed by incubation with BV510 Ki67 antibody (Biolegend) for 15 min at RT. A minimum of 20,000 events were recorded for analysis. Compensation was computed using BD FACS diva software using single staining of Comp ebeads (eBioscience) and checked manually. Fluorescent minus one control (antibody cocktail containing all antibodies except one) was used for gating. Data were analyzed by flowjo© software. All expression was classified as positive immunophenotype if the frequency of positive cells was more than 30% of the parent population.

CCR4^−^, CCR4^+^ CD26^+^, and CCR4^+^ CD26^−^ subsets of CD4 T cells were sorted on BD Aria III and collected into staining buffer. Purity >90% was confirmed by reanalyzing the sorted cells.

### HTLV‐1 PVL quantification

Genomic DNA was extracted from PBMCs and sorted CD4 T‐cell subsets using QIAamp DNA mini kit (Qiagen). Proviral load in PBMCs was determined by real‐time polymerase chain reaction as previously described 20. Tarl 2 was used for the standard curve to quantify PVL in the sorted cell populations as it has a single copy of HTLV‐1 provirus integrated in the host cell genome.

### Integration site analysis

The assay is described in detail in supplementary text. Briefly, genomic DNA is digested with four restriction enzymes, AclI, ApaLI, EcoRI, and PciI, (New England Biolab, UK) that do not cut HTLV‐1, generating DNA fragments with an average size of 1000 bp. Digestion is followed by ligation of a linker (with the same overhang sequence) to the digested DNA. The sequences flanking the integration sites of the virus are then amplified using two HTLV‐1‐specific primers and two vectorette‐specific primers. The amplicons are detected by electrophoresis in a 1.5% agarose gel. The assay is conducted in triplicate.

A clone with a large number of cells will be detected in each of the triplicates and is reported as a dominant clone. A small clone will be detected inconsistently. A malignant clone may be detected as a single amplicon or as a dominant amplicon on a polyclonal background as shown in Figure S1.

### Statistics

Statistical analysis was performed using Graphpad Prism software©. Correlation significance between two continuous variables was determined by nonparametric Spearman test. The significance of difference in continuous variable between two and multiple groups was determined by Mann–Whitney U tests and Kruskal–Wallis test with Dunn posttest analysis, respectively. The significant difference in contingency variable was determined by chi‐square test.

## Results

### CD4^+^ CCR4^+^ CD26^−^ as the immunophenotype of HTLV‐1‐infected cells

**Figure 1:**
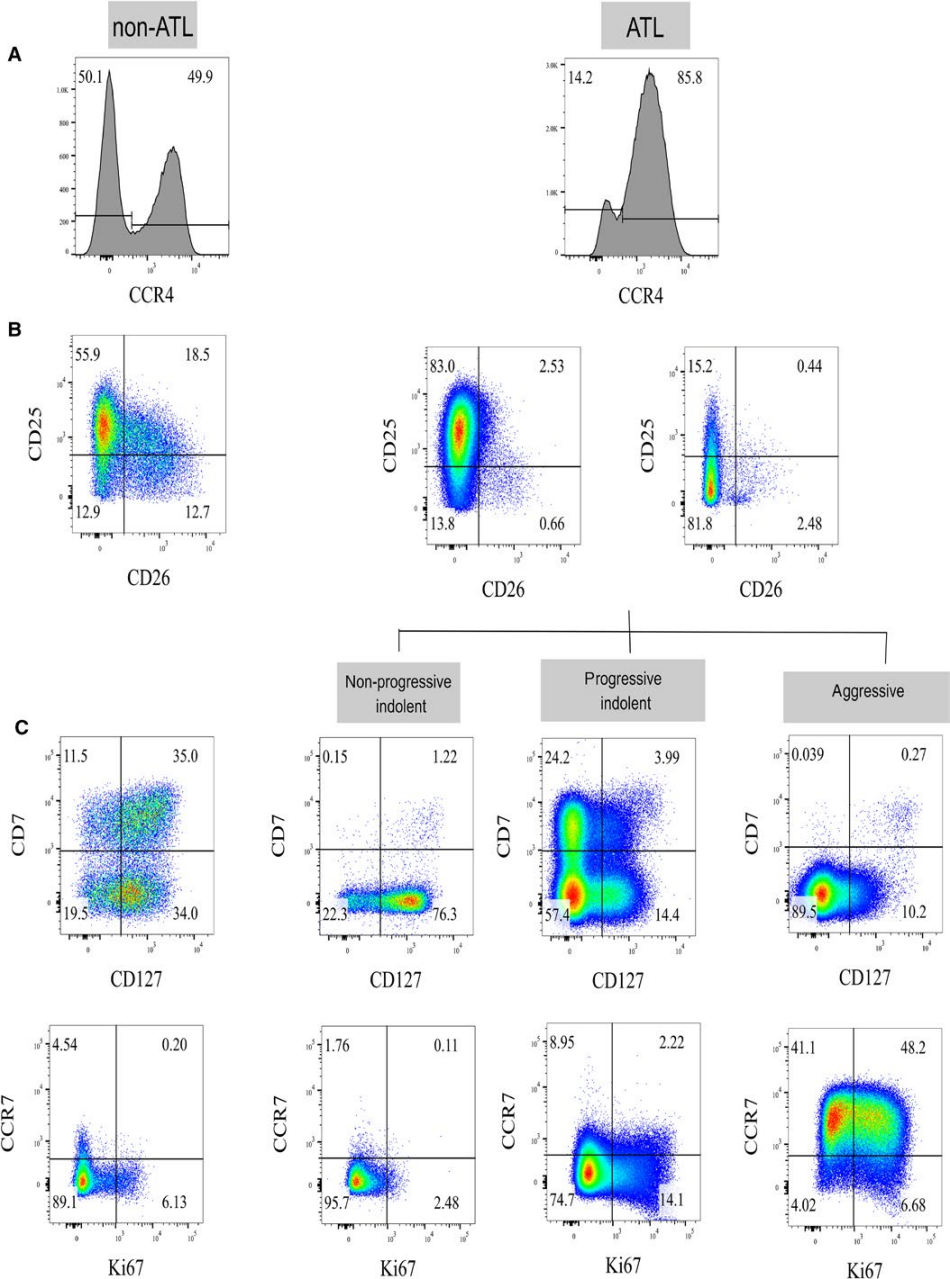
Representative plots of immunophenotyping in non‐ATL and ATL human T‐lymphotropic virus type 1 (HTLV‐1) infection: (A) Histogram plots of CCR4 expression in CD4^+^ T cells (A), pseudocolor plots of CD26 and CD25 expression within CD4^+^ CCR4^+^ T cells (B), and CD7, CD127, CCR7, and Ki67 expression within CD4^+^ CCR4^+^ CD26^−^ T cells (C) in patients with non‐ATL and ATL HTLV‐1 infection.

**Figure 2:**
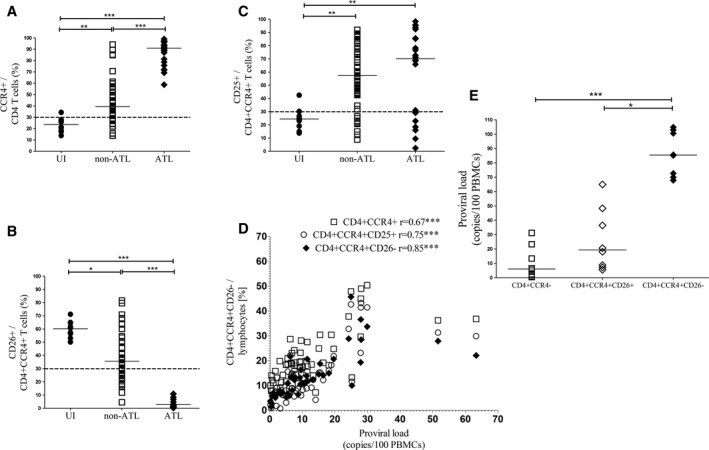
Immunophenotype of human T‐lymphotropic virus type 1 (HTLV‐1)‐infected cells: Aligned column plots showing significant stepwise change in CCR4^+^ (A) and CD26^+^ (B) within CD4^+^ and CD4^+^ CCR4^+^ T cells, respectively, from uninfected individuals (UI) through patients with non‐ATL to ATL HTLV‐1 infection. All ATL had CD4^+^ CCR4^+^ CD26^−^ immunophenotype. (C) CD25^+^ cells were higher in patients with non‐ATL and ATL compared to UI, but there was no difference between non‐ATL and ATL. (D) XY plots showing proviral load (PVL) had stronger correlation with CD4^+^ CCR4^+^ CD26^−^ than CD4^+^ CCR4^+^ and CD4^+^ CCR4^+^ CD25^+^ T cells in non‐ATL HTLV‐1 infection. (E) Aligned column plots showing significantly higher PVL within sorted CCR4^+^ CD26^−^ compared to CCR4^−^ and CCR4^+^ CD26^+^ CD4 T‐cell subsets. The bar represents median values and the dotted line the cutoff for positive expression. Statistical analysis: Kruskal–Wallis test with Dunn posttest, 95% confidence interval, and Spearman test, respectively. * denotes *P* < 0.05, ** denotes *P* < 0.01, *** denotes *P* < 0.001. r= Spearman correlation coefficient.

CD4 T cells were gated using the gating strategy shown in Figure S2. Representative plot of expression of CCR4, CD25, and CD26 within CD4^+^ T cells is shown in Figure 1. The frequency of CCR4^+^ in CD4^+^ T cells showed a significant stepwise increase from uninfected individuals through non‐ATL to ATL as shown in Figure 2A. The increase in CD4^+^ CCR4^+^ T cells was predominantly due to CD26^−^ and CD25^+^ cells as shown in Figure 2B and 2C, respectively. CD26^+^ cell relative frequencies showed a significant stepwise decrease from uninfected individuals through patients with non‐ATL to ATL. CD25^+^ cell relative frequencies were higher in patients with non‐ATL and ATL than uninfected individual, but there was no difference between non‐ATL and ATL.

The PVL is the measure of infected cells in PBMCs by HTLV‐1 DNA copy numbers quantified per 100 PBMC. The PVL showed stronger correlation with CD4^+^ CCR4^+^ CD26^−^ than CD4^+^ CCR4^+^ and CD4^+^ CCR4^+^ CD25^+^ T cells in patients with non‐ATL HTLV‐1 infection as shown in Figure 2D. There was no difference in the relative frequencies of these cells between ACs and patients with HAM (data not shown). To confirm CD4^+^ CCR4^+^ CD26^−^ as the main reservoir of infection burden in patients with non‐ATL HTLV‐1 infection, CCR4^−^, CCR4^+^ CD26^+^, and CCR4^+^ CD26^−^ subsets of CD4 T cells were sorted from eight patients (Four ACs and four patients with HAM) and PVL was significantly higher in CCR4^+^ CD26^−^(Median:85.5%) compared to CCR4^−^(Median: 6.2%) and CCR4^+^ CD26^+^(Median:19.4%) subsets of CD4 T cells as shown in Figure 2E.

All patients with ATL had CCR4^+^ (≥30% CCR4^+^ in CD4 T cells) and CCR4^+^ CD26^−^ (<30% CD26 expression in CD4^+^ CCR4^+^ T cells) immunophenotype, whereas only 77% had CCR4^+^ CD25^+^ (≥30% CD25 expression in CD4^+^ CCR4^+^ T cells) immunophenotype. Five patients with ATL (three indolent and two aggressive) had CD4^+^ CCR4^+^ CD25^−^ immunophenotype. There was no difference in lymphocytosis or other disease characteristic between patients with CD25^+^ and CD25^−^ immunophenotype. These data suggest CD4^+^ CCR4^+^ CD26^−^ is a better marker of infected cells than CD4^+^ CCR4^+^ CD25^+^ in both non‐ATL and ATL HTLV‐1 infection and that it is not specific to the leukemic (ATL) cells in ATL.

### Diagnosis and prognostication of ATL

CD7, Ki67, CD127, and CCR7 expression within the CD4^+^ CCR4^+^ CD26^−^ T cells was determined as shown in Figure 1.

#### CD7^−^ phenotype as diagnostic marker of ATL

**Figure 3:**
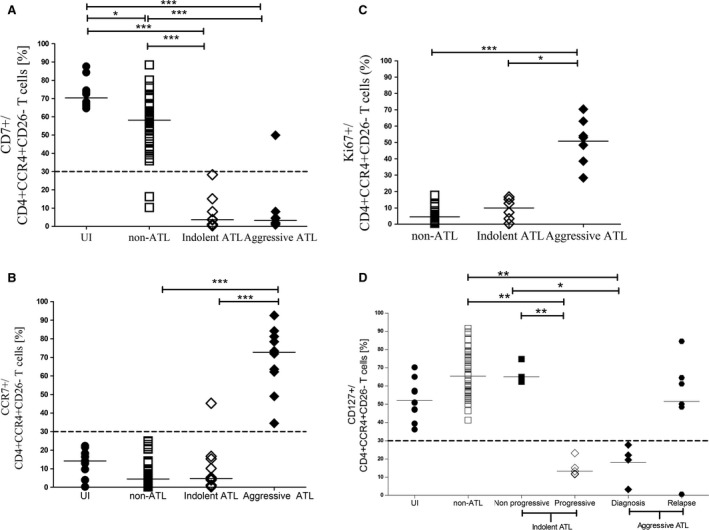
Immunophenotype for diagnosis and prognosis of ATL: Gated on CD4^+^ CCR4^+^ CD26^−^ cells (A) Aligned column plots showing significant stepwise decrease in CD7^+^ cells (A) from UI through patients with non‐ATL to ATL HTLV‐1 infection. CCR7^+^ (B) and Ki67 (C) cells were significantly higher in patients with aggressive ATL compared to non‐ATL and indolent ATL. CD127^+^ cells (D) were significantly lower in indolent ATL with progressive lymphocytosis compared to those with nonprogressive lymphocytosis and non‐ATL HTLV‐1 infection. The bar represents median values and the dotted line the cutoff for positive expression. Statistical analysis: Kruskal–Wallis test with Dunn posttest, 95% confidence interval. * denotes *P* < 0.05, ** denotes *P* < 0.01, *** denotes *P* < 0.001.

There was a stepwise decrease in CD7^+^ cell relative frequencies from uninfected individuals through patients with non‐ATL to ATL as shown in Figure 3A. Ninety‐six percent of patients with non‐ATL HTLV‐1 infection had CD7^+^ immunophenotype (≥30% CD7^+^ within CD4^+^ CCR4^+^ CD26^−^ T cells), whereas 95% patients with ATL had CD7^−^ immunophenotype (<30%). These observations suggest that CD4^+^ CCR4^+^ CD26^−^ CD7^−^ phenotype has high sensitivity and specificity to differentiate ATL and non‐ATL HTLV‐1 infection.

#### CCR7^+^ immunophenotype as marker of aggressive ATL

The CCR7^+^ cell relative frequencies within CD4^+^ CCR4^+^ CD26^−^ T cells was significantly higher in patients with aggressive ATL than those with indolent ATL and non‐ATL as shown in Figure 3B. All patients with aggressive ATL had a CCR7^+^ (≥30% CCR7^+^ expression in CD4^+^ CCR4^+^ CD26^−^ T cells) immunophenotype, whereas 92% patients with indolent ATL, 100% with non‐ATL, and 100% uninfected individuals had CCR7^−^ immunophenotype.

#### Ki67 expression for prognosis

Ki67 expression within CD4^+^ CCR4^+^ CD26^−^ T cells was determined in 22 patients with non‐ATL and 13 patients with ATL HTLV‐1 infection. Ki67 expression was significantly higher in patients with aggressive ATL compared to indolent ATL and non‐ATL as shown in Figure 3C. This observation confirmed that the leukemic cells in aggressive ATL had higher proliferation rates than those in cases of indolent ATL and also showed proliferation of leukemic cells in indolent ATL is similar to infected cells in non‐ATL.

#### CD127^−^ immunophenotype as a marker of indolent ATL with progressive lymphocytosis

The CD127^+^ cell relative frequencies within CD4^+^ CCR4^+^ CD26^−^ T cells were significantly lower in patients with indolent ATL and progressive lymphocytosis than those with non‐ATL HTLV‐1 infection and indolent ATL with nonprogressive lymphocytosis as shown in Figure 3D. All uninfected individuals, patients with non‐ATL HTLV‐1 infection, and indolent ATL with nonprogressive lymphocytosis (*n* = 5) had a CD127^+^ immunophenotype (≥30% CD127^+^ expression in CD4^+^ CCR4^+^ CD26^−^ T cells), whereas patients with indolent ATL with progressive lymphocytosis (*n* = 5) had a CD127^−^ immunophenotype. Five out of six patients with aggressive ATL and samples collected at relapse following systemic therapy (two chemotherapy and three AZT + INF combination, respectively) had CD127^+^ immunophenotype while all four at diagnosis were CD127^−^ immunophenotype.

#### “ATL‐like” cells in high‐risk non‐ATL HTLV‐1 infection

**Figure 4:**
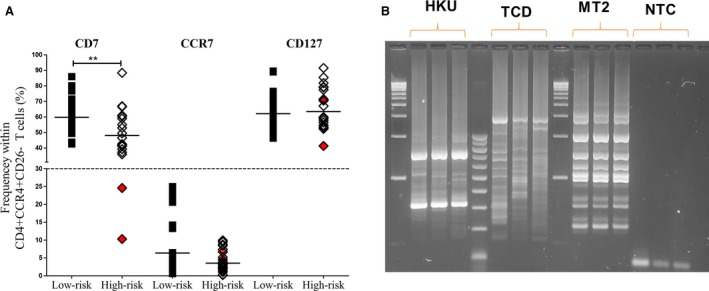
“‘ATL‐like” cells in non‐ATL human T‐lymphotropic virus type 1 (HTLV‐1) infection. (A) Aligned plots of CD7, CCR7, and CD127 expression in patients with low‐risk (PVL <10%) and high‐risk (PVL≥10%) HTLV‐1 infection. Two patients with non‐ATL HTLV‐1 infection (HKU and TCD) had “ATL‐like” (CD7^−^) immunophenotype as highlighted in red color. Statistical analysis: Spearman test. * denotes *P* < 0.05, ** denotes *P* < 0.01, *** denotes *P* < 0.001. r= correlation coefficient. (B) Integration site analysis by vectorette showing presence of dominant band. MT2 and NTC are positive and negative control, respectively.

Patients with high‐risk non‐ATL HTLV‐1 infection (PVL>10%, *n* = 20) had significantly higher frequency of CD7^−^ within CD4^+^ CCR4^+^ CD26^−^ T cells than those with low risk as shown in Figure 4A. This suggest that patient at high risk of ATL have an increased frequency of “ATL‐like” cells. There was no difference in CD127 and CCR7 expression between low risk and high risk. CCR7^+^ and CD127^−^ immunophenotype was not seen in non‐ATL.

Two (10%) patients with high risk had CD7^−^ immunophenotype similar to ATL as highlighted in red color in Figure 4A. These patients had normal CD4 T‐cell counts and no evidence of progression to overt ATL after 71 and 18 months of follow‐up, respectively. Clonality by integration site analysis showed the presence of dominant bands as shown in Figure 4B. These patients had CCR7^−^ CD127^+^ immunophenotype and low Ki67 expression similar to indolent ATL with a nonprogressive course. These observations suggest that dominance of CD7^−^ is a pre‐ATL event, while CD127^−^ and CCR7^+^ are later leukemogenesis events.

### Longitudinal study in patient with malignant evolution

**Figure 5:**
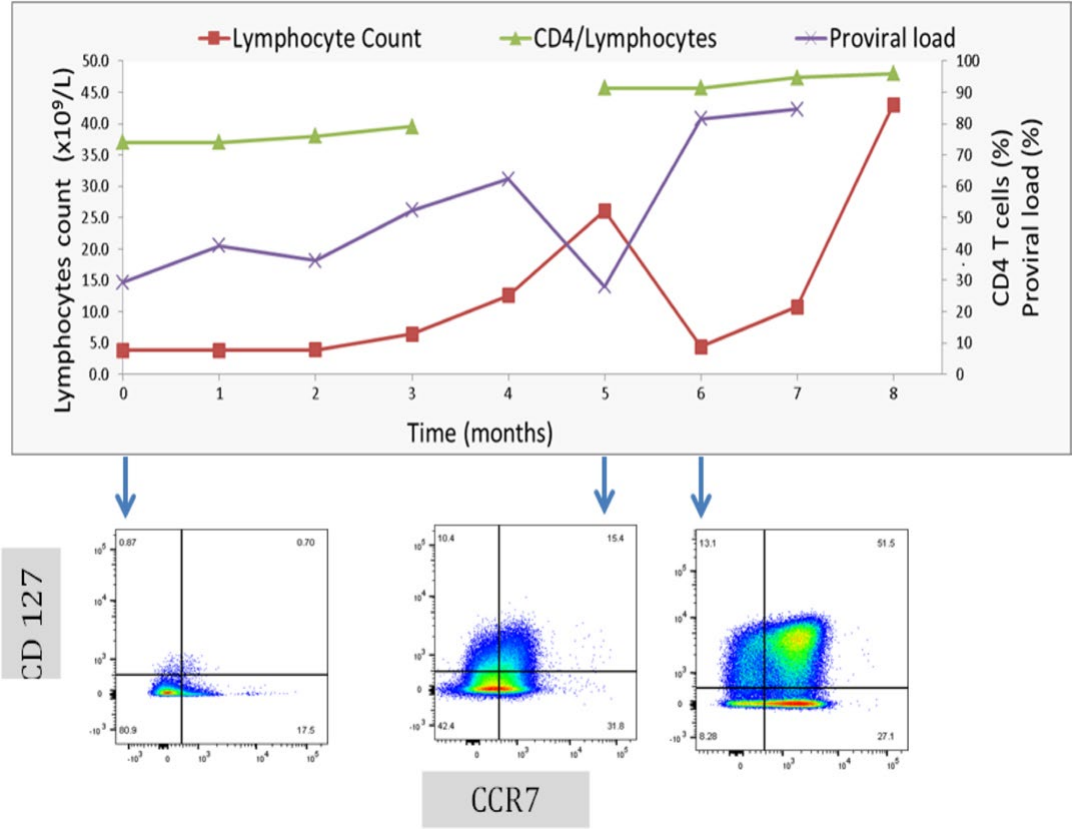
Longitudinal study of malignant evolution in ATL. Trends in lymphocytes count, frequency of CD4^+^ T cells, and proviral load in a patient with ATL (LGL). LGL presented with indolent ATL transforming to aggressive ATL and suffered early relapse with pseudocolor plots of CCR7 (x axis) and CD127 (y axis) expression in CD4^+^ CCR4^+^ CD26^−^ CD7^−^ T cells shows CD127^−^ CCR7^−^, CD127^−^ CCR7^+^ and CD127^+^ CCR7^+^ at indolent with progressive lymphocytosis, aggressive transformation, and posttreatment stage, respectively.

Figure 5 shows the malignant evolution in a 57‐year‐old female of Afro‐Caribbean origin who presented with unfavorable indolent ATL (multiple skin lesions and lymphocytosis). Her disease progressed to acute ATL (progressive lymphocytosis and renal infiltration) 4 months later. ZDV+IFN treatment resulted in transient partial response, followed by progressive disease, and she died 5 months following treatment initiation. Immunophenotyping showed CD4^+^ CCR4^+^ CD26^−^ CD7^−^ immunophenotype throughout the course of disease. CD127 and CCR7 expression varied with the stages of disease progression: CD127^−^ CCR7^−^ immunophenotype, markers of indolent ATL with progressive lymphocytosis were present at first presentation, CD127^−^ CCR7^+^ immunophenotype was detected at aggressive transformation and the CD127^+^ CCR7^+^ immunophenotype was noted at the treatment refractory disease stage.

## Discussion

ATL has a highly variable clinical course and there is a need for better markers for both diagnosis and prognosis. Immunophenotyping is widely available and routinely used for diagnosis and classification of hematological malignancies. The currently used immunophenotypic criterion for ATL diagnosis is CD3^+^ CD4^+^ CD25^+^ CD7^−^. However, this definition has significant shortcomings: CD4^+^ CD25^+^ CD7^−^ is also the immunophenotype of infected cells in non‐ATL HTLV‐1 infection, and ATL with a CD25^−^ or CD7^+^ immunophenotype has been commonly reported; so these markers lack sensitivity and specificity for the diagnosis of ATL 17, 21, 22, 23.

The aim of this study was to characterize the immunophenotypic difference between non‐ATL and ATL HTLV‐1 infection. These data suggest that CD4^+^ CCR4^+^ CD26^−^ is a better marker of infected cells than CD4^+^ CCR4^+^ CD25^+^ and that the CD4^+^ CCR4^+^ CD26^−^ CD7^−^ immunophenotype is a more sensitive and specific marker than CD4^+^ CD25^+^ CD7^−^ to differentiate ATL and non‐ATL HTLV‐1 infection. However, this phenotype is also frequently seen in other cutaneous T‐cell malignancy 24 and does not differentiate ATL and non‐ATL T‐cell malignancies. PVL and viral integration site analysis is most helpful in these instances 20, 25. Both CCR4^+^ 16, 26 and CD26^−^ immunophenotype 18 have been shown previously to be associated with both non‐ATL and ATL HTLV‐1 infection. Downregulation of CD26 within CD4^+^ CCR4^+^ CD25^+^ T cells in patients with indolent ATL has been reported 27, but our data suggest this to be a feature of HTLV‐1 infection per se.

ATL occurs almost exclusively in patients with established high PVL and our data suggest that these high‐risk patients have an increase in “ATL‐like” cells compared to low‐risk patients. A small subgroup (10%) of high‐risk patients had dominance of “ATL‐like” cells within infected cells similar to indolent ATL. These patients had dominant clones similar to the description of a “Pre‐ATL” state which has been shown to have a higher incidence of ATL progression 28. Although no progression to clinical ATL disease was seen in patient with “ATL‐like” immunophenotype, these cells were similar to nonprogressive indolent ATL and follow‐up is short. Our approach of using immunophenotyping has an advantage over clonality studies by integration site analysis (which is not widely available) or T‐cell receptor analysis (where it is difficult to interpret in the context of chronic infection 29. Furthermore, immunophenotyping provides additional prognostic information about the ATL cells in addition to its clone size.

The prognostic value of subtyping chronic ATL into favorable and unfavorable subtypes was not confirmed as 30% of unfavorable ATL had a nonprogressive course. Similar discrepancies have been recently reported by the Japanese Lymphoma Group 8. Indolent ATL with progressive lymphocytosis requiring systemic treatment had a CD127^−^ CCR7^−^ immunophenotype, whereas both indolent ATL with stable lymphocytosis and non‐ATL HTLV‐1 infection had a CD127^+^ CCR7^−^ immunophenotype. Variable expression of CD127 (the interleukin 7 receptor *α* chain) in CD4^+^ T cells of patients with ATL has been reported, although the clinical and molecular significance is not known 30. We have shown the prognostic value of CD127 in indolent ATL. CD127 expression can be influenced by receptor shedding, translation, transcription, or mutations 31. We did not find a relationship between cellular CD127 expression and either cellular CD25 and FOXP3 or plasma concentration of IL2, IL7, and IL10 (data not shown). Further study into the molecular factors driving the specific immunophenotype associated with non‐ATL and ATL HTLV‐1 infection is needed for a full understanding of the leukemogenesis process in ATL.

Our data highlight the prognostic value of the CCR7^+^ immunophenotype as it was associated with aggressive, treatment refractory disease. CCR7 expression within CD4^+^ T cells in patients with ATL has been shown to correlate with lymph nodal enlargement 15 and CCR7 mutations are common in ATL 32. The CCR7^+^ immunophenotype has been shown to be associated with higher infiltrative properties 33. CCR7 is also a potential target for treatment by anti‐CCR7 antibody 34. Systemic treatment refractory stage was associated with CD127^+^ CCR7^+^ suggesting this immunophenotype is especially refractory to therapy.

The main limitation of our study is a relatively small patient cohort. However, ATL is a rare disease outside Japan and clinical studies on non‐Japanese patients are few. There are significant differences in presentation and management between Japan and other regions 35, 36, 37, 38. Our cohort of patients with HTLV‐1 infection, of mainly Afro‐Caribbean ancestry, is the largest in Europe. The incidence of ATL in UK is approximately 15 cases annually. We studied patient samples collected over more than 10 years in order to understand the leukemogenic process in non‐Japanese patients. Our novel findings allow risk stratification of patients with HTLV‐1 infection and ATL using a widely available technique. This will facilitate confirmation in a larger cohort and translation into clinical practice.

**Figure 6:**
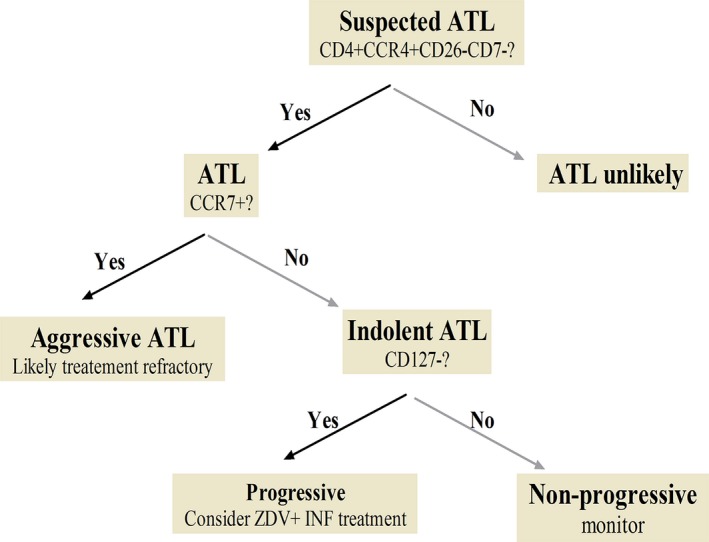
Proposed flow diagram for diagnosis and prognostication of ATL.

The flow diagram shown in Figure 6 is suggested for the diagnosis and prognostication of ATL. Patients with suspected ATL (high‐risk non‐ATL HTLV‐1 infection or suspicious clinical features) should be examined for the presence of the CD4^+^ CCR4^+^ CD26^−^ CD7^−^ immunophenotype using a gating strategy to determine CCR4^+^ (≥30% CCR4 expression in CD4 T cells), CD26^−^ (<30% CD26 expression in CD4^+^ CCR4^+^ T cells), and CD7^−^ (<30% CD7 expression in CD4^+^ CCR4^+^ CD26^−^ T cells). If this immunophenotype is present, CD127 and CCR7 expression should be determined. The CD127^+^ CCR7^−^ (>30% CD127 and <30% CCR7 expression within CD4^+^ CCR4^+^ CD26^−^ T cells) immunophenotype is being associated with indolent ATL and a stable course which can be monitored 3–6 monthly, whereas patients with a CD127^−^ CCR7^−^ immunophenotype are predicted to have a progressive course and treatment with ZDV+IFN should be considered. Aggressive ATL had a CD127^+/−^ CCR7^+^ immunophenotype and immediate systemic treatment should be considered. However, these patients were refractory to both ZDV+IFN and chemotherapy in our cohort and should be offered clinical trials of novel agents if available.

## Conflict of Interest

The authors declared no conflicting financial interest.

## Supporting information


**Table S1**. Fluorochrome‐labeled antibody used.
**Data S1.** Method.
**Figure S1**. Vectorette assay. Vectorette assay showing multiple small clones with no dominant band (A), dominant band (B), and dominant band on polyclonal background (c), respectively. All samples tested in triplicates.
**Figure S2**. Gating strategy for CD4^+^ T cells.
