# Supplementary material for: Risk stratification of adult T‐cell leukemia/lymphoma using immunophenotyping
Source: Cancer Med. 2016 Dec 30;6(1):298–309. doi: 10.1002/cam4.928 (PMC5269699; doi:10.1002/cam4.928)
Supplement: Supplementary file 1 — Table S1. Fluorochrome‐labeled antibody used. Data S1. Method. Figure S1. Vectorette assay. Vectorette assay showing multiple small clones with no dominant band (A), dominant band (B), and dominant band on polyclonal background (c), respectively. All samples tested in triplicates. Figure S2. Gating strategy for CD4+ T cells. [file CAM4-6-298-s001.docx]

**Supplementary Table 1: Flurochrome labelled antibody used**

| Marker | Antibody | Supplier |
| --- | --- | --- |
| CD3 | Alexa Fluor 700 anti-human CD3 | BioLegend, San Diego, USA |
| CD4 | Fluorescein isothiocyanate anti-human CD4 |  |
| CD8 | Brilliant Violet 605 anti-human CD8a |  |
| CD25 | Phycoerythrin (PE) anti-human CD25 |  |
| CD26 | Allophycocyanin (APC) anti human CD26 |  |
| CD127 | Brilliant Violet 421 anti-human CD127 |  |
| CCR4 | PerCP/Cy5.5 anti-human CD194 (CCR4) |  |
| CCR7 | Brilliant Violet 650/Brilliant Violet 510/APC-Cyanine (CY) 7 anti-human CD197 |  |
| Ki67 | Brilliant Violet 510/APC anti-human Ki-67 |  |
| Viability stain | Zombie near infra-red Fixable Viability Kit |  |
| CD7 | CD7 PE-CY 7 anti-human CD7 | eBioscience, San Diego, USA |

**Supplementary method**

**Vectorette assay**

**Introduction**

The integration site analysis is performed to detect HTLV-1 integration site host flanking sequences and clonal expansion of HTLV-1 bearing T-cells. The technique involve ligation of a vectorette linker to fragmented genomic DNA, which enables PCR amplification between known sequences in the viral LTR and the linker using two specific proviral primers, BIO2 and BIO3. This method is adopted to investigate clonality in ATL and non ATL HTLV-1 infection.

**Method**

A linker mediated PCR assay was developed based on the Vectorette^TM^ Genomic Systems (Sigma –Aldrich, USA). The method enable PCR amplification of DNA sequences which lie between a single known primer and a nearby restriction site. The linkers (or vectorette units) consist of a pair of annealed oligonucleotides that contain two regions of complementary nucleotide sequence flanking a non-complementary segment. The 5' terminus of one of these complementary regions is phosphorylated and displays a restriction enzyme-specific sticky end that permits ligation of the linkers (vectorette units) to both ends of a restriction fragment. Primers VP1 and VP2, directed toward the phosphorylated end, have most of their sequences in the non-complementary region of the vectorette. These oligonucleotides cannot function as PCR primers on vectorette units without synthesis of a complementary strand from a primer located in the ligated DNA. Thus, although vectorette units will ligate to themselves and to all restriction fragments with matching ends, only DNA flanked by a specific primer and a vectorette will be amplified.

**Construction of the Vectorette unit**: four vectorette units, one for each restriction site chosen, were constructed. For each vector the upper-arm oligo was combined with the corresponding lower arm oligo at a final concentration of 10 µM, in annealing buffer (100 mM Tris-HCl, pH 7.5, 1 M NaCl, 10 mM EDTA). The oligo solution was heated to a temperature of 94° C for 10 minutes and then removed from the heating block and allowed to cool slowly to room temperature (at least one hour). The 10 µM annealed vector solutions were stored as a stock solution at -20°C.

The Vectorette system consists of three key steps:

1. Digestion of genomic DNA with restriction enzymes

2. Ligation of the Vectorette units to the restriction digested DNA fragments, generating a Vectorette library.

3. PCR using one primer directed at the Vectorette unit and a primer targeting the known DNA sequence. This allows PCR of a fragment of DNA between the known sequence and the restriction site used to cut the target DNA.

**Digestion:** One microgram of DNA is digested using four restriction endonucleases (ApaL1, Acl1, EcoR1 and Pci1 (New England Biolabs Inc.), none of which cut within the HTLV-1 genome. A DNA library with an average fragment length of 1000 base pairs was generated, thus minimising bias arising from preferential PCR amplification of shorter products.

**Ligation:** One μl of each Vectorette units is ligated to the digested DNA sample (diluted 10 times with water) using 1 unit of T4 DNA ligase (New England Biolabs, UK). The reaction is incubated at room temperature for 15 minutes and the ligated DNA (diluted 1/5) is used as a template for the Linker mediated PCR.

**Linker mediated PCR**: Two step touchdown PCRs are carried out using one primer identical to a sequence in the mismatched region of the vectorette (VP1 for the first round and VP2 for the second round) and one primer specific for the HTLV-1 5´ long terminal repeat (BIO2 for the first round and BIO3 for the second round). For a 25 µl PCR reaction the following reagents were combined in a sterile microcentrifuge tube:

First round: one µl of Vectorette library, 2.5 µl of 10x PCR buffer, 0.5 µl of 10mM dNTPs mix, 0.25 µl of 10µM Vectorette primer 1(VP1) , 2.5 µl of 10µM HTLV-1 specific primer (BIO2), 1.25 µl of 1U/µl Taq DNA polymerase (JumpStart REDAccuTaq LA DNA polymerase, Sigma-Aldrich, St. Louis, MO). Reaction is amplified using the following conditions: 94°C, 15 seconds, 70°C, 5 minutes – 4 cycles; 94°C, 15 seconds, 68°C, 5 minutes – 25 cycles; 68°C, 10minutes – 1cycle 4°C . PCR product is diluted 1 in 5 before second round is performed.

Second round: one µl of ligated DNA, 2.5 µl of 10x PCR buffer, 0.5 µl of 10mM dNTPs mix, 1 µl of 10µM Vectorette primer 2(VP2) , 1 µl of 10µM HTLV-1 specific primer (BIO3), 1.25 µl of 1U/µl Taq DNA polymerase. Reaction is amplified using the following conditions: 94°C, 15 seconds, 70°C, 5 minutes – 4 cycles; 94°C, 15 seconds, 68°C, 5 minutes – 40 cycles; 68°C, 10minutes – 1cycle 4°C .

Samples are analysed in triplicate to distinguish dominant bands (present in all replicates) from the polyclonal background (bands not present in all replicates) as shown in supplementary figure 1.

If a clone has a large number of cells, it will be detected consistently in every replicate that uses these PCR-based assays and will be considered a dominant clone . If a clone has only a small number of cells, it will be detected inconsistently and will be considered a minor clone.

We defined Dominant band a band present consistently in all the replicates of the test, and divided the clonal patterns in: monoclonal, oligoclonal and polyclonal. Subjects were considered positive if at least one Dominant band was present.

**Oligo’s**

| Name | Sequence 5’-3’ |
| --- | --- |
| Acl-Upper | P-CGTTAAGGAGAGGACGCTGTCTGTCGAAGGTAAGGAACGGACGAGAGAAGGGAGAG |
| Acl-Lower | CTCTCCCTTCTCGAATCGTAACCGTTCGTACGAGAATCGCTGTCCTCTCCTTAA |
| Apa-Upper | P-TGCACAAGGAGAGGACGCTGTCTGTCGAAGGTAAGGAACGGACGAGAGAAGGGAGAG |
| Apa-Lower | CTCTCCCTTCTCGAATCGTAACCGTTCGTACGAGAATCGCTGTCCTCTCCTTG |
| Eco-Upper | P-AATTCAAGGAGAGGACGCTGTCTGTCGAAGGTAAGGAACGGACGAGAGAAGGGAGAG |
| Eco-Lower | CTCTCCCTTCTCGAATCGTAACCGTTCGTACGAGAATCGCTGTCCTCTCCTTG |
| Pci-Upper | P-CATGTAAGGAGAGGACGCTGTCTGTCGAAGGTAAGGAACGGACGAGAGAAGGGAGAG |
| Pci-Lower * | CTCTCCCTTCTCGAATCGTAACCGTTCGTACGAGAATCGCTGTCCTCTCCTTA |
| VP1 | TCTCCCTTCTCGAATCGTAACCGTTCGTTCGTAC |
| BIO2 | CTGTTCTGCGCCGTTACAGATCGA |
| VP2 | CGAATCGTAACCGTTCGTACGAGAATCGCT |
| BIO3 | CCTTTCATTCACGACTGACTGCCG |

**
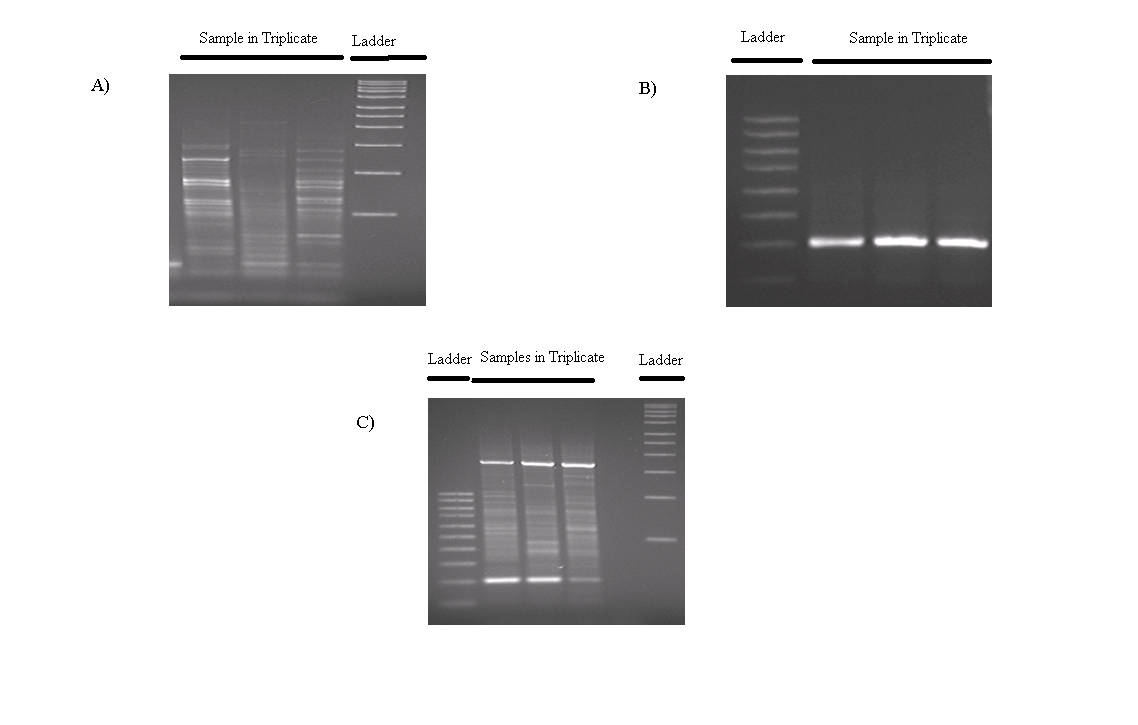
**

**Supplementary Figure 1: Vectorette assay. Vectorette assay showing multiple small clones with no dominant band (A), dominant band (B) and dominant band on polyclonal background (c) respectively. All samples tested in triplicates.**


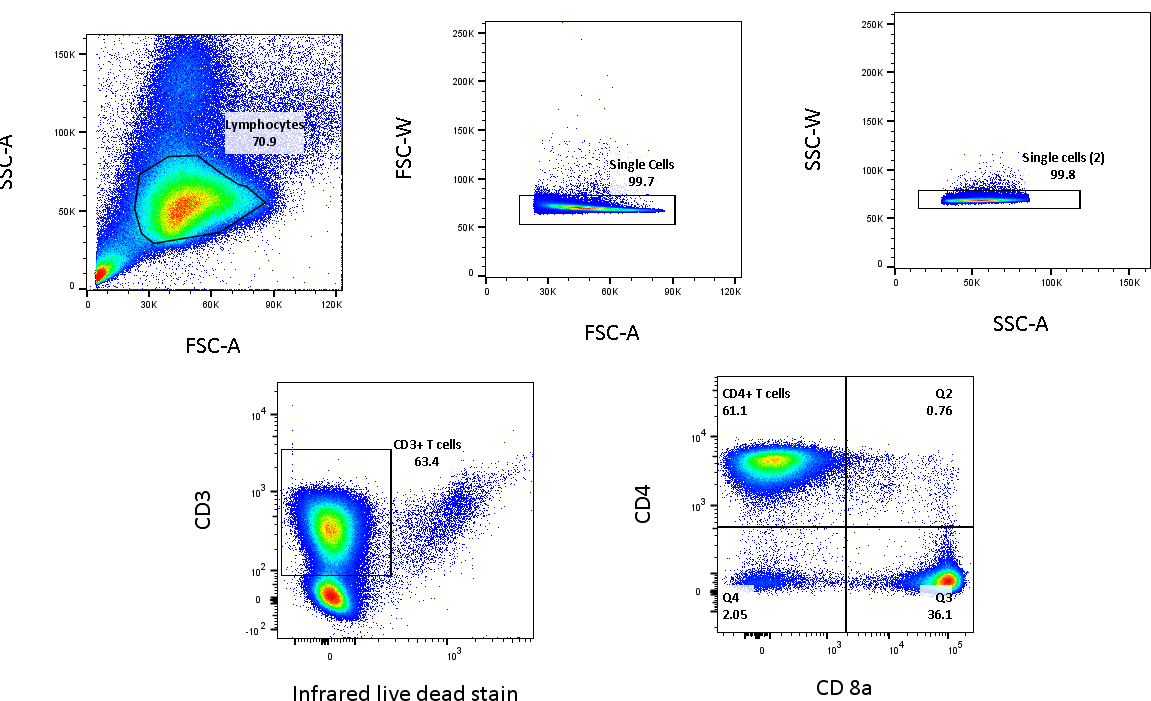


**Supplementary Figure 2:** Gating strategy for CD4+ T cells.
